# Tetrahydroxystilbene Glucoside (TSG) Restores the Effect of Transient Hypoxia on Reperfusion Injury in Senescent H9c2 Cells by Regulating Mitochondrial Energy Metabolism

**DOI:** 10.1155/2018/2545024

**Published:** 2018-12-13

**Authors:** Yin Cheng, Jia Song, Pengfei Hu, Yun Zhu

**Affiliations:** ^1^Department of Emergency, Zhejiang Hospital, Hangzhou, 310012, China; ^2^Department of Emergency, The Second Affiliated Hospital of Zhejiang Chinese Medical University, Hangzhou, 31005, China; ^3^Department of Cardiology, The Second Affiliated Hospital of Zhejiang Chinese Medical University, Hangzhou, 31005, China

## Abstract

Tetrahydroxystilbene glucoside (TSG) is extracted from a famous Chinese herbal medicine which is widely used as an antiaging agent in history. Lots of studies gave evidence that TSG exhibited benefits to brain, like improvement of learning and memory and synaptic plasticity. Moreover, the polyphenolic structure of TSG enables its capability to prevent cerebral ischemia/reperfusion injury (IRI) by reducing apoptosis and ROS/RNS generation. Due to its antioxidant profile, TSG had been demonstrated to alleviate cardiac toxicity by regulating biochemical indexes and ROS. However, whether TSG exhibited cardioprotective effects via mitochondrial energy metabolic functions, which played crucial role in IRI, remained unclear. Here, we used an* in vitro* aging model of cardiomyocytes to evaluate the effects of TSG on transient hypoxia-pretreated hypoxia/reoxygenation (H/R) injury and mitochondrial energy metaolism. Our results showed that TSG enhanced cardioprotective effect of transient hypoxia on H/R by reducing excessive ROS production and calcium overloading. Significant improvements of mitochondrial respiratory functions and ketone body metabolism elucidated that TSG restored the effect of transient hypoxia on H/R injury in aging cardiomyocytes via upregulating mitochondrial energy metabolism.

## 1. Introduction

Ischemic preconditioning (IPC), targeting to mitochondrial ATP-sensitive potassium channel (K_ATP_ channel), was one of the effective clinical/experimental interventions to prevent ischemia/reperfusion injury (IRI) [1]. Mechanistic studies had verified selectively activating K_ATP_ channel by pharmacological intervention performed the approximately effects of IPC, but not in aging hearts [2]. One of natural compounds had been discovered to restore the cardioprotective effects of IPC in aging hearts was resveratrol with polyphenol structure [3]. Resveratrol was natural compound derived from grapes and red wine, performing the anti-aging and anti-oxidant profiles [4–6]. Taking these aspects as consideration, another natural compound tetrahydroxystilbene glucoside (TSG), derived from traditional Chinese herb* Polygonum multiflorum* with polyphenolic structure, probably have the ability to restore IPC's effects in aging hearts [7, 8].

The neuroprotective effects of TSG had shown that TSG significantly reduced cognitive impairments and improved the hippocampal synaptic plasticity [7, 9], and further improved brain memory by activating SIRT1 expression and phosphorylation of ERKs, CaMKII [10]. Depending on these activities, TSG intervention restored learning memory in APP transgenic mice, a mouse model of Alzheimer's disease, and increased neuron survival in Parkinson's mice via PI3K/Akt signaling [11, 12]. Based on the beneficial effects of TSG on brain, Chen's group found pretreatment of TSG prevented cerebral ischemia injury via inhibition of ROS/RNS generation, involving JNK and NF-*κ*B signaling [13]. All of these studies indicated TSG probably have beneficial effect on cardiac IPC against prolonged I/R injury in aging hearts.

Here, we applied an* in vitro* model of I/R to evaluate whether TSG could restore the effect of transient hypoxia against H/R injury in senescent H9c2 cardiomyocytes. We stepped forward to detect mitochondrial energy metabolism, including mitochondrial respiration and ketone body metabolism, and uncovered the role of both energetic metabolisms in regulation of TSG in senescent H9c2 cells. Finally, we demonstrated TSG restored the effect of transient hypoxia on H/R injury by regulating mitochondrial OXPHOS and ketone body metabolism.

## 2. Materials and Methods

### 2.1. Cell Culture and Pharmacological Interventions

H9c2 cardiomyocyte cell line was purchased from the American Type Culture Collection (CRL1446, ATCC, USA) and cultured in full Dulbecco's Modified Eagle Medium (DMEM) composed of 10% fetal bovine serum (FBS). TSG (Sigma-Aldrich, St. Louis, MO, USA) was given 24 h before transient hypoxia and H/R. H9c2 cells were divided into 8 groups: (i) control, cultured in normoxia; (ii) H/R alone; (iii) transient hypoxia; (iv) transient hypoxia + H/R; (v) TSG alone; (vi) TSG + H/R; (vii) transient hypoxia + TSG; and (viii) transient hypoxia + TSG + H/R

### 2.2. *In Vitro* Model of Senescence, H/R and Transient Hypoxia

Senescent H9c2 cells were induced by D-galactose (D-gal) with 20 g/L for 60 h after cell adherence and all the H9c2 cells used in this study were senescent. H9c2 cells hypoxia was induced by replacing the normoxia medium with no glucose DMEM and placing cells into a chemical hypoxia system (BD, USA). After 12 h hypoxia, H9c2 cells were subjected to reoxygenation by replacing with cultural medium for 24 h. Transient hypoxia was performed 2 h before H/R injury.

### 2.3. Cell Viability and Mitochondrial Viability

Cell viabilities of H9c2 cells after different treatments were determined by using 3-(4,5-dimethylthiazol-2-yl)-2,5-diphe-nyltetrazolium bromide (MTT) assay purchased from Sigma-Aldrich (St. Louis, MO, USA). After reoxygenation, cultural medium containing MTT (500 *μ*g/ml) was added to the plates (100 *μ*l/well) and incubated for 4 h at 37°C. Then 10% SDS-HCl solution was added to solubilize formazan crystals formed in viable cells. The absorbance was measure optical density at 570/650 nm by multimode plate reader (SpectraMax Paradigm, Molecular Devices, USA). Cell viability of group without any treatment was considered as 100%.

The mitochondrial viability was detected by the mitochondrial viability assay kit (ab129732, Abcam, USA). After 4 h dye staining, 96-well microplate was shaken gently for 5 min, then the fluorescence was detected at 550/590 nm in dark. Mitochondrial viability of group without any treatment was considered as 100%.

### 2.4. Measurements of Reactive Oxygen Species (ROS) and Calcium (*Ca*^2+^)

ROS and calcium were measured by BD FACSAria III flow cytometer (BD, USA) using the DCFDA ROS probe (Invitrogen, USA) and Fluo-4, AM (Invitrogen, USA) fluorescent probe, respectively, according to the manufactory's methods. Briefly, cells were digested by Trypsin-EDTA and resuspended with PBS for washing. Then, cells were incubated with DCFDA or Fluo-4 probe for 30 min at 37°C in dark. The ROS production and calcium concentration in each group were indicated by intensity of green fluorescence (Ex 488nm/ Em 535nm). Fluorescent intensity of group without any treatment was considered as 100%.

### 2.5. Measurements of Oxygen Consumption Rate (OCR) and Respiratory Function

Mitochondrial OCR and respiratory function were detected by using the XF Cell Mito Stress Test with Seahorse XFp Extracellular Flux Analyzer (Seahorse Bioscience, USA). H9c2 cells were seeded on the XFp cell culture miniplates (5000/well) and the sensor cartridge was hydrated in a non-CO2 incubator at 37°C one day before assay. After H/R, the port A on the sensor cartridge was injected with 1.5 *μ*M oligomycin (complex V inhibitor), 2 *μ*M FCCP, and 0.5 *μ*M rotenone/antimycin A (inhibitors of complex I and complex III) were loaded to ports A, B, and C, respectively. After sensor calibration, the sensor was put on the cell plate immediately and detected by Mito Stress Test. OCR, ATP production, basal respiration, and maximal respiration were calculated in according to protocol and normalized to protein concentration in each well.

### 2.6. RNA Extraction, Reverse Transcription, and Quantitative Real-Time PCR

Cellular RNA was extracted by using FavorPrep™ RNA purification kit (Favorgen Biotech Corp.). Cells were lysed in 300 *μ*l of lysis buffer and total RNA was isolated on spin columns following the manufacturer's instructions. After that, RNA was eluted with 30 *μ*l water without RNase and purified RNA (1 *μ*g) was reversal transcribed to 20 *μ*l system. Synthesized cDNA was then detected by Quantitative real-time PCR, performing with the real-time PCR system using the FastStart Universal SYBR-Green kit according to the manufacturer's instructions. Primer sequences directed against ACAT1 and OXCT1 were listed in Table 1 for mRNA quantification, and *β*-actin was used as an endogenous control. The PCR mixture was comprised with 10 *μ*l SYBR mix, 1 *μ*g template, ddH2O, 0.5 *μ*l forward primers, and 0.5 *μ*l reverse primers, making up to 20 *μ*l. The procedure of RCR included 50°C for 2 min and 95°C for 10 min and was followed by 45 cycles of 95°C for 15 s and 60°C for 60 s. Gene expression level was based on the comparative CT value and normalized with *β*-actin.

### 2.7. Measurements of Cellular BHB and ACAC

Cellular BHB was detected by using a Cayman's Colorimetric Assay Kit. Briefly, cells were washed with PBS for 1 time and digested by trypsin and then collected by centrifuging at 2,000 g for 10 minutes. Pellets were resuspended with 1 ml cold assay buffer (1×) and supersonicated with 1 second bursts for 20 times and centrifuged at 10,000 g for 10 minutes. Then, the pellets were resuspended in 1 ml of cold assay buffer for further detection. 50 *μ*l of developer solution was added to 50 *μ*l of samples and plate was incubated in the dark for 30 minutes and read plate with the absorbance at 450 nm.

The concentration of ACAC was detected after different treatments by using a Colorimetric Assay Kit (Abcam, UK). Cells pellets were collected and resuspended with ddH_2_O as above. Sample volumes (10-100 *μ*l) were adjusted to 110 *μ*l by ddH_2_O, 80 *μ*l ACAC assay buffer and 10 *μ*l ACAC substrate were added and mixed well. Plate was incubated at 25°C for 15 min and the values of absorbance at OD 550 nm were measured in a kinetic mode.

### 2.8. Measurements of Cellular Acetyl-CoA

Cellular acetyl-CoA concentrations were determined using a commercial Kit (Abnova, TaiWan). H9c2 cells were collected and super-sonicated rapidly on ice and centrifuged at 10000 × g, 4°C. Supernatants were stayed on ice for 5 min and neutralized pH to 6-8 with 3 M KHCO_3_ and diluted volume to 50 *μ*l. Then added reaction mix with 50 *μ*l to samples and incubated for 10 min at 37°C. The plate was measured with fluorescent detection using Ex/Em= 535/589 nm. Fluorescent intensity of group without any treatment was considered as 100%.

### 2.9. Statistics

Data were analyzed using Student t-test between 2 groups by GraphPad Prism 7.0. All data were presented as the means ± SEM.* P*<0.05 was represented as statistical significance.

## 3. Results

### 3.1. TSG Restored the Effect of Transient Hypoxia on Reperfusion Injury in Senescent H9c2 Cells

**Figure 1:**
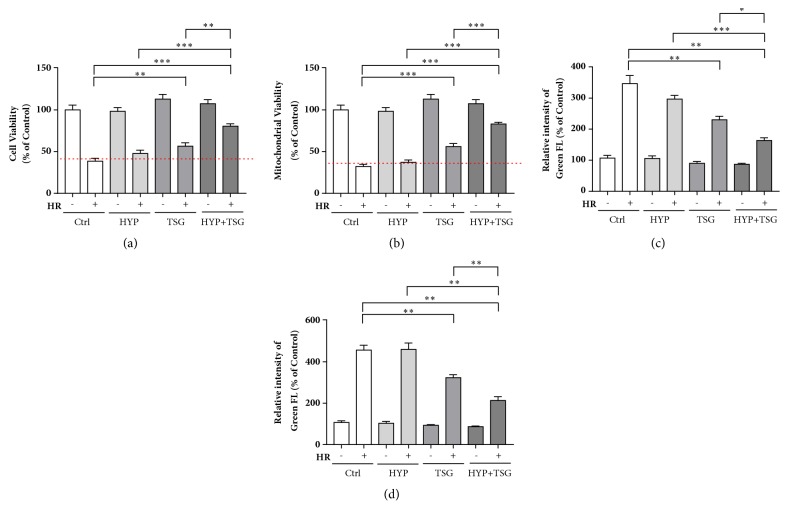
TSG restored protective effect of transient hypoxia (TH) on senescent H9c2 cells against HR injury. (a) Effect of TH and TSG on cell viability in senescent H9c2 cells against HR injury determined by MTT assay. Data (n=5) were shown as the mean ± SEM, *∗p*<0.05 and *∗∗p*<0.01, *∗∗∗p*<0.001. (b) Effect of TH and TSG on mitochondrial viability in senescent H9c2 cells against HR injury determined by Mitochondrial Viability assay. Data (n=5) were shown as the mean ± SEM, *∗∗∗p*<0.001. (c, d) Effects of TH and TSG on ROS production and Ca^2+^ concentration in senescent H9c2 cells against HR injury determined by flow cytometry, respectively, stained by H_2_DCFDA and Fluo-4, AM. Data (n=3) were shown as the mean ± SEM, *∗p*<0.05, *∗∗p*<0.01, *∗∗∗p*<0.001.

TSG had been reported to alleviate aging-associated diseases and prevent I/R injury. Whether TSG can restore the effect of transient hypoxia on long-term H/R injury in senescent H9c2 cells was tested in our study. We firstly evaluated the cell viability and mitochondrial viability in senescent cells. H/R injury significantly decreased cell viability in control group and transient hypoxia (HYP) did not show any cardioprotective effect. Excitingly, combined treatment of HYP and TSG showed an increasing cell viability and mitochondrial viability comparing with control group, indicating TSG could restore the cardioprotective effect of HYP. Moreover, HYP plus TSG group displayed higher activity comparing with HYP only group and TSG only group, respectively (Figures 1(a) and 1(b)).

To further demonstrate TSG's ability to restore HYP's cardioprotective effect on senescent H9c2 cells, ROS, and calcium overloading, two crucial markers for H/R injury were detected by flow cytometry. The cellular concentrations of ROS and calcium increased significantly after H/R injury (≥ 3 times) and HYP treatment had no effect on the excessive productions of ROS and calcium, while combined treatments of HYP and TSG evidently reduced cytotoxicity by decreasing ROS production and calcium overloading, compared with HYP only groups and TSG only groups (Figures 1(c) and 1(d)).

Taken together, TSG restored the cardiac beneficial effect of transient hypoxia in senescent H9c2 cells via reduction of cellular ROS, calcium overloading, and mitochondrial toxicity, based on its antiaging profile.

### 3.2. TSG Restored the Effect of Transient Hypoxia on Reperfusion Injury in Senescent H9c2 Cells via Mitochondrial Respiratory Function

Transient hypoxia stimuli activated mitochondrial respiratory function against mitochondrial turnover and mitochondria-mediated cell apoptosis and organ damage. However, mitochondrial functions were disrupted in aging progress, especially in energy-consumption organ, such as heart. Due to antiaging profile of TSG, we hypothesized TSG pretreatment had ability to alleviate biological alterations of aging, which probably can restore the effect of transient hypoxia by regulation of mitochondrial respiratory function.

**Figure 2:**
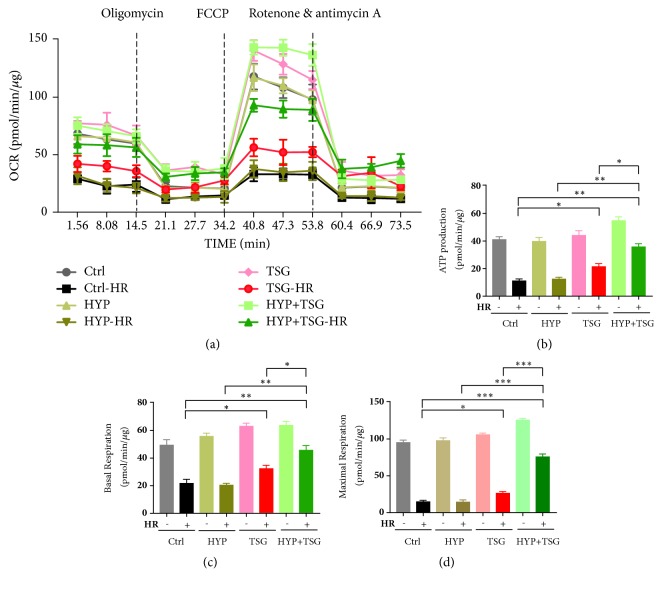
TSG restored protective effect of transient hypoxia (TH) on senescent H9c2 cells against HR injury by regulating mitochondrial OXPHOS. (a) Effect of TH and TSG on OCR in senescent H9c2 cells against HR injury determined by mitochondrial respiration kit. (b, c, d) Effect of TH and TSG on ATP production, basal respiration, and maximal respiration in senescent H9c2 cells against HR injury. Data (n=3) were shown as the mean ± SEM, *∗p*<0.05, *∗∗p*<0.01 and *∗∗∗p*<0.001.

We tested living cell oxygen consumption rate (OCR) by XP Extracellular Flux Analyzer and results showed that HYP did not enhance OCR when cells were exposed to H/R injury. Pretreatment of HYP plus TSG enhanced OCR against H/R injury in senescent H9c2 cells (Figure 2(a)). We further analyzed respiration-related parameters, ATP production, basal respiration, and maximal respiration, to understand TSG's effect in detail. Consistent with the result of OCR curve, HYP could not upregulate ATP production, basal respiration, and maximal respiration in senescent cells against H/R. Meanwhile, TSG only group and HYP plus TSG group significantly upregulated ATP production, basal respiration, and maximal respiration, the latter group displaying more effective mitochondrial respiratory functions than TSG only group and restored the effect of HYP (Figures 2(b), 2(c), and 2(d)).

Collectively, TSG restored the cardiac beneficial effect of transient hypoxia in senescent H9c2 cells via enhancement of mitochondrial respiratory functions, based on its antiaging profile.

### 3.3. TSG Restored the Effect of Transient Hypoxia on Reperfusion Injury in Senescent H9c2 Cells via Ketone Body Metabolism

**Figure 3:**
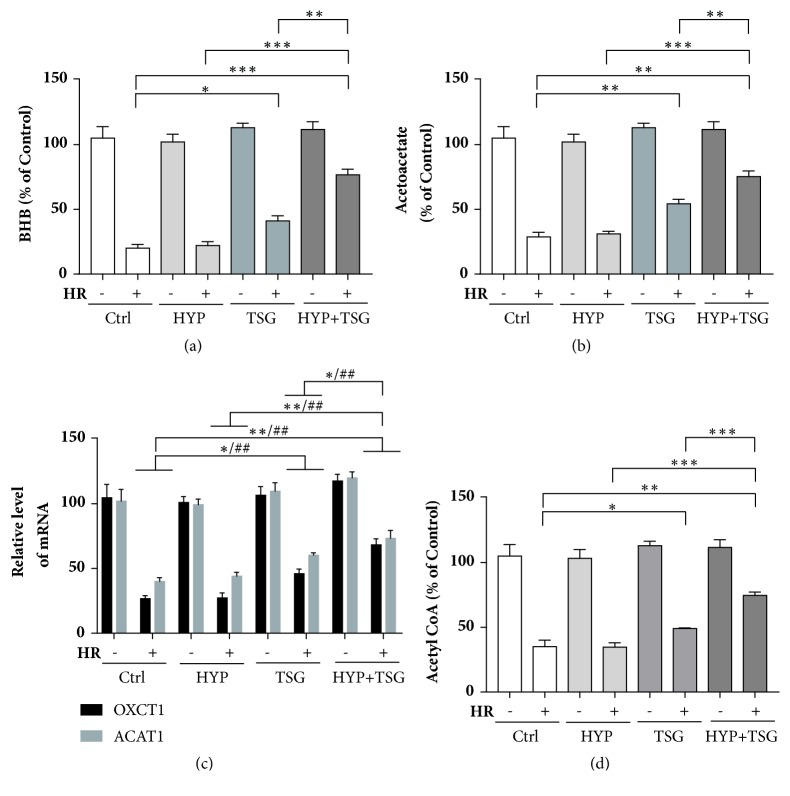
TSG restored protective effect of transient hypoxia (TH) on senescent H9c2 cells against HR injury by regulating ketone body metabolism. (a, b) The concentration of *β*-hydroxybutyrate (BHB) and acetoacetate (ACAC)in cell extractions. Data (n=3) were presented as mean ± SEM, *∗p*<0.05, *∗∗p*<0.01, *∗∗∗p*<0.001. (c) mRNA levels of OXCT1 and ACAT1 were quantified by real-time RT-PCR. Data (n=3) were presented as mean ± SEM. OXCT1: *∗p*<0.05 and *∗∗p*<0.01; ACAT1: ^##^*p* < 0.01. (d) The concentration of acetyl CoA in cell extractions. Data (n=3) were presented as mean ± SEM, *∗p*<0.05, *∗∗p*<0.01 and *∗∗∗p*<0.001.

Mitochondrial OXPHOS was important for cardiac energy demand even under reperfusion period, in which peroxidative injury damaged mitochondrial respiration. Therefore, other energy metabolic utilizations, like ketone body, were essential for cardiac survival and working well. We evaluated the level of two major components of ketone body, *β*-hydroxybutyrate (BHB), and acetoacetate (ACAC). Firstly, we found that the levels of BHB and ACAC significantly decreased after H/R injury in the control group, suggesting that ketone body metabolism was damaged. HYP hypoxia could not raise the levels of BHB and ACAC; however pretreatment of TSG had the ability to increase their levels in senescent cells. Interestingly, in group of HYP combined with TSG, the levels of BHB and ACAC evidently increased comparing with the single treatment of HYP or TSG, respectively, suggesting TSG restored the HYP's effect on ketone body in senescent cardiomyocytes (Figures 3(a) and 3(b)).

Crucial regulators of ketone body metabolism were OXCT1 and ACAT1, whose mRNA level was analyzed in different treatments. After H/R, the mRNA level of both proteins decreased significantly in control group, as well as in HYP group, indicating loss of cardioprotective effect of HYP in senescent cardiomyocytes. Single treatment of TSG evidently upregulated mRNA expression levels of OXCT1 and ACAT1 against senescence. Importantly, TSG pretreatment increased the levels of OXCT1 and ACAT1 comparing with HYP and TSG single treatment group, respectively, suggesting the restoration of HYP' ability in cardioprotection against H/R injury (Figure 3(c)).

Acetyl-CoA was the final product in ketone body utilization in heart to supply the material for TCA cycle. We evaluated the concentration of Acetyl-CoA to examine whether the upregulations of ketone body and its relative enzymes performed effective functions in cardiac energy supplementary when facing H/R injury. The results were exactly as what we thought that combined treatment of HYP and TSG significantly increased Acetyl-CoA comparing with HYP only group and TSG only group, respectively (Figure 3(d)).

In summary, TSG pretreatment restored cardioprotective effects of HYP against H/R injury via upregulations of mitochondrial respiration and ketone body metabolism in senescent cardiomyocytes.

## 4. Discussion

Our study amplified the knowledge of TSG application in cardiac injury, especially in senescent cardiomyocytes, based on its antiaging profiles. We also gave insight into functional regulation of these polyphenols or compounds with polyphenol structure in restoration of transient hypoxia, or even hypoxic/ischemic preconditioning. We firstly used* in vitro *model of aging by long-term treatment of D-gal according to previous studies [14–17]. Then we found transient hypoxia could not increase cell viability and mitochondrial viability against H/R injury in senescent H9c2 cells, which was consistent with previous reports [1, 3, 15, 18]. In the group of TSG single treatment, TSG increased cell and mitochondrial viability decreased ROS production and calcium overloading against H/R injury, comparing with cells in control group, suggesting TSG's original antiaging and prevention from H/R injury. When cells were treated by TSG plus HYP, significant enhancements of cell and mitochondrial viability were found with reductions of ROS production and calcium overloading under H/R injury.

As a compound extracted from an antiaging Chinese herb, TSG could alleviated aging-associated phenotypes in lots of studies [11, 19–21]. However, the relationship between TSG and mitochondria was not uncovered, considering the crucial role of mitochondria in aging progress and aging-related diseases. Our study evaluated mitochondrial respiratory function and found TSG single treatment had ability to enhance OCR and ATP production in senescent cardiomyocytes against H/R injury. Furthermore, TSG restored the effect of transient hypoxia to protect senescent cells from H/R injury via upregulation of OCR, ATP production, and basal and maximal respiration. That suggested that TSG, containing a polyphenol structure, probably retain mitochondrial viability and respiratory function in aging cardiomyocytes for restoration of transient hypoxia's effect on long-term H/R injury.

Ketone body, composed with *β*-hydroxybutyrate (BHB), acetoacetate (AcAc), and acetone, derived from liver and played an important role in energy supply when cell experienced glucose and oxygen deprivation in heart and brain [22]. Hepatic ketogenesis applied acetyl-CoA to produce ketone body and delivered to heart trough blood. In heart, ketone body was transferred to acetyl-CoA by regulation of OXCT1 and ACAT1 for utilization of TCA cycle in mitochondria [23]. Therefore, we detected the levels of BHB and ACAC, two major components of ketone body, and mRNA expressions of OXCT1 and ACAT1 in our study. Interesting results were that TSG single treatment significantly increased levels of BHB and ACAC and also the mRNA expressions of OXCT1 and ACAT1 in cells exposed to H/R. More importantly, combined treatments of TSG and HYP exhibited the higher levels of these parameters, suggesting TSG restored HYP's effect in senescent cardiomyocytes against H/R injury, in partly, by upregulating ketone body metabolism. Finally, the level of acetyl-CoA increased in TSG plus HYP group, indicating more production of acetyl-CoA for TCA cycle.

**Figure 4:**
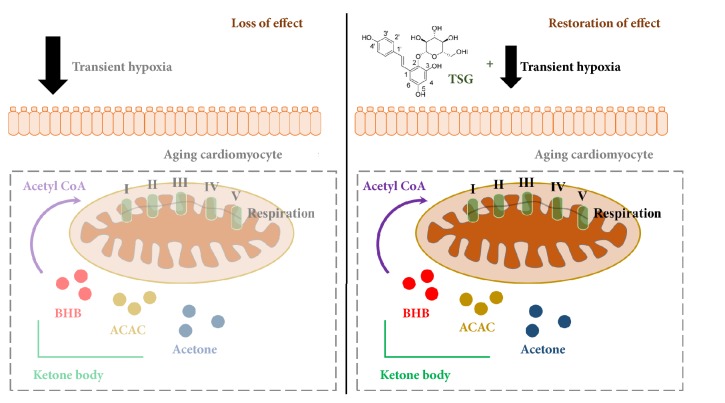
Schematic representation raised the effect of Tetrahydroxystilbene glucoside (TSG) on restoration of transient hypoxia's cardioprotective effect in aging cardiomyocytes. Left graph represented cells without TSG treatment: transient hypoxia cannot upregulate mitochondrial respiration and ketone body metabolism in aging cardiomyocytes; right graph represented cells with TSG treatment: after TSG treatment, transient hypoxia restores the upregulation of mitochondrial respiration and ketone body metabolism in aging cardiomyocytes.

In conclusion, TSG assisted transient hypoxia to restore its protective effect on senescent cardiomyocytes against H/R injury via reduction of ROS production and calcium overloading, enhancement mitochondrial respiratory function and ATP production, and ketone body metabolism (Figure 4).

## Figures and Tables

**Table 1:** Primer sequences of ACAT1 and OXCT1.

Name	Forward	Reverse
ACAT1	5′-ATGGCTGCCCTGGCGGTTCTA-3′	5′-CTACAGCTTCTCAATCAGCAC-3′
OXCT1	5′-CTGGAGTTTGAGGACGGCAT-3′	5′-TCCGCATCAGCTTCGTCTTT-3′
*β*-Actin	5′-GCTACAGCTTCACCACCACA-3′	5′-ATCGTACTCCTGCTTGCTGA-3′

## Data Availability

The data used to support the findings of this study are included within the article in Figures 1–3.
